# Assessment of peripheral dose as a function of distance and depth from cobalt-60 beam in water phantom using TLD-100

**DOI:** 10.1186/s43046-024-00227-1

**Published:** 2024-06-24

**Authors:** Habib Ahmad, Javaid Ali, Khalil Ahmad, Ghufran Biradar, Ashfaq Zaman, Yasir Uddin, Muhammad Sohail, Shahid Ali

**Affiliations:** 1 Swat Institute of Nuclear Medicine, Oncology & Radiotherapy (SINOR) Cancer Hospital, Saidu Sharif Swat, KPK Pakistan; 2 Larkana Institute of Nuclear Medicine and Radiotherapy (LINAR) Cancer Hospital, Larkana, Sindh Pakistan; 3 Pakistan Institute of Nuclear Science and Technology (PINSTECH), Islamabad, Pakistan; 4 Royal College of Nursing, Saidu Sharif, Swat, KPK Pakistan; 5 International Collaborative Laboratory of 2D Materials for Optoelectronics Science and Technology of Ministry of Education, Institute of Microscale Optoelectronics, College of Electronics and Information Engineering, Shenzhen University, Shenzhen, 518060 China; 6 Department of Physics, University of Peshawar (UOP), Peshawar, KPK Pakistan

**Keywords:** Peripheral dose, TLD-100, Co-60 dosimetry, Depth dose, Penumbra

## Abstract

**Background:**

Innovations in cancer treatment have contributed to the improved survival rate of cancer patients. The cancer survival rates have been growing and nearly two third of those survivors have been exposed to clinical radiation during their treatment. The study of long-term radiation effects, especially secondary cancer induction, has become increasingly important. An accurate assessment of out-of-field/peripheral dose (PDs) is necessary to estimate the risk of second cancer after radiotherapy and the damage to the organs at risk surrounding the planning target volume. This study was designed to measure the PDs as a function of dose, distances, and depths from Telecobalt-60 (Co-60) beam in water phantom using thermoluminescent dosimeter-100 (TLD-100).

**Methods:**

The PDs were measured for Co-60 beam at specified depths of 0 cm (surface), 5 cm, 10 cm, and 15 cm outside the radiation beam at distances of 5, 10, and 13 cm away from the radiation field edge using TLD-100 (G1 cards) as detectors. These calibrated cards were placed on the acrylic disc in circular tracks. The radiation dose of 2000 mGy of Co-60 beam was applied inside 10 × 10 cm^2^ field size at constant source to surface distance (SSD) of 80 cm.

**Results:**

The results showed maximum and minimum PDs at surface and 5 cm depth respectively at all distances from the radiation field edge. Dose distributions out of the field edge with respect to distance were isotropic. The decrease in PDs at 5 cm depth was due to dominant forward scattering of Co-60 gamma rays. The increase in PDs beyond 5 cm depth was due to increase in the irradiated volume, increase in penumbra, increase in source to axis distance (SAD), and increase in field size due to inverse square factor.

**Conclusion:**

It is concluded that the PDs depends upon depth and distance from the radiation field edge. All the measurements show PDs in the homogenous medium (water); therefore, it estimates absorbed dose to the organ at risk (OAR) adjacent to cancer tissues/planning target volume (PTV). It is suggested that PDs can be minimized by using the SAD technique, as this technique controls sources of scattered radiation like inverse square factor and effect of penumbra up-to some extent.

## Background

Radiation therapy (RT) is an effective treatment for cancer and the objectives of radiation therapy is to give the maximum radiation dose to the cancer tissues/target and minimum or no dose to the surrounding healthy tissues [1–3]. The number of radiotherapy cases are increasing day by day [4]. With the development/innovations in radiotherapy treatment modalities like 3D CRT, IMRT, VMAT SRS, SBRT, and IGRT with dose escalation and conformity of the target volume, there is increase in cure rats and patient surveillance [5]. The cancer surveillance has been increased, and nearly two third of these survivors are exposed to clinical radiation during their treatment [6]. The study of long-term radiation effects, especially the second cancer induction has become increasingly important. As many secondary cancers appears far from the target volume/PTV, therefore, the dose out of the field at peripheries (PD) should always be considered for theoretical assessment of secondary cancer risk [3, 7–9]. During radiotherapy treatment with high-energy photon beams, a small fraction of the delivered dose is absorbed a few centimeters away from the treatment beam/field [10]; this dose is known as peripheral dose (PD) and compared to high doses within the target volume. The associated cancer risk is likely to be much lower but not insignificant [11]. The risk of secondary cancer associated with low doses of ionizing radiation especially appears in long term survivors is gaining new interest every day [12]. Most of the secondary cancer within the margins of the treatment field (from 2.5 cm in to 5 cm out) has received a dose less than 6 Gy [13]. There is a 40% increase in solid tumor in lung after radiotherapy in prostate where the lung received a dose from 0.5 to 1.0 Gy [14]. Therefore, there is no dose that is regarded as safe. It is important to assess PDs to radiosensitive tissue/organs, such as the breast, gonads, and the thyroid to determine the possible risk of late effects, such as secondary cancers that could appear in long-term surviving patients (e.g., pediatric patients) [15]. PDs may also cause some radiation-induced diseases like cataract, infertility, lung fibrosis, and myocarditis and also contraindicated in pregnant patients [16]. In general, it is of extreme importance to calculate the PDs down to the level of 0.1% of the central axis maximum dose (*d*_max_), and its determination has been the subject of extensive investigation [17]. The photon PDs has three sources: (a) leakage through the head shielding and the collimation systems; (b) scattering from the head and secondary collimators, and (c) scattering inside the patient/phantom [18]. Moreover, in the case of Co-60 unit, the penumbra due to source size is also an additional component of PDs. The scattering in the patient is the dominant source of the PD in regions close to the irradiated volume. However, its relative contribution to the total PDs rapidly decreases for further distances from the treatment edge, leaving collimator scattering and leakage as the predominant dose sources in those regions. At considerable distances, leakage is the only relevant dose source [19, 20]. Therefore, medical physicist is responsible to ensure that radio-sensitive tissues outside of the radiation beam do not receive doses approaching their tolerance levels. Detailed knowledge and accurate estimation of the magnitude and spatial distribution of the PDs is necessary, as this can be used in retrospective studies examining possible correlation between radiotherapy dose and secondary cancer incidence in radiotherapy patients [17]. It is also the responsibility of medical physicist to correctly estimate the radiation absorbed doses to OAR due to PDs and make accurate radiotherapy treatment plan [16].

There are no commercial treatment planning systems (TPSs) designed/available for the precise calculation of the PD and its significant deviations, compared to measurements and Monte Carlo (MC) simulations [21–23]. Several published mathematical models exist for estimating secondary cancer induction probability as a function of the radiation dose, which should count with an accurate out-of-field dose distribution received by the patient during RT [24–26]. The software Peri dose was probably the first attempt to calculate scattered dose outside the primary beam for individual treatments. However, it was only designed to be used for rectangular fields [10]. A simple and flexible analytical model for PDs estimation was also implemented into a computer program termed PERIPHOCAL, correctly predicted the PDs inside a humanoid phantom irradiated with IMRT and VMAT techniques. It presents, however, two main limitations: (a) the model was trained using only a few measurements points placed inside a humanoid phantom, and (b) it is one dimensional, i.e., it assumed that the organs were described only by the *z* coordinate of the organ and its length along the craniocaudal direction [27, 28]. Using complex mathematical functions to represent the physics behind each process and calculate the three peripheral dose components separately, a different approach to model peripheral dose was applied by Hauri et al. [29]. Other recently published models also considered calculating each contribution of the PDs separately. They did calculations in water cylinders with fast computation times but at the price of needing several fitting coefficients. Despite their high accuracy, the main disadvantage of those approaches is their complexity, which makes the clinical application very cumbersome [30, 31]. An attempt/study is to figure out the PD distribution outside of the radiation field at surface and depths using Co-60 teletherapy unit in our institute has been carried out.

In this study, TLD-100 (G1 cards), as a very effective tool for dose measurement [32], was used to measure the PDs received outside the treatment field at 5 cm, 10 cm, and 13 cm away from the field edge at surface (0 cm), 5 cm, 10 cm, and 15 cm depths in the water phantom. Also, the isotropic distribution of dose inside the tissue equivalent medium (water) from the source was carried out. Furthermore, the scattered to primary dose ratios at all depths and distances from the radiation field edges were estimated. Modern Co-60 teletherapy machines with the MLCs and 100 cm SSD are frequently in use for IMRT treatment. Intensity-modulated radiation therapy (IMRT) withCo-60 teletherapy [33, 34] can be suitable for complex superficial anatomic sites, and it can minimize the incidence of radiation toxicity in proximal organ at risk volume [35, 36]. Integrating technologies like multileaf collimator in Co-60 teletherapy [37–39] units can facilitate automated treatment [40, 41]. It is therefore important for medical physicists to consider the role of Co-60 teletherapy in advanced technologies like IMRT [41–43]. Co-60-based radiation therapy continues to play a significant role in not only developing countries where access to RT is extremely limited but also in industrialized countries [33, 34, 39, 44].

Therefore, from this study, the behavior of the Co-60 gamma ray beam inside the homogenous scattering medium was observed. By adding some useful techniques (factors), one can evaluate/estimate the radiation dose received by OAR, if the distance of the OAR is known outside the treatment field/volume, which is the significance of the study.

## Methods

**Fig. 1:**
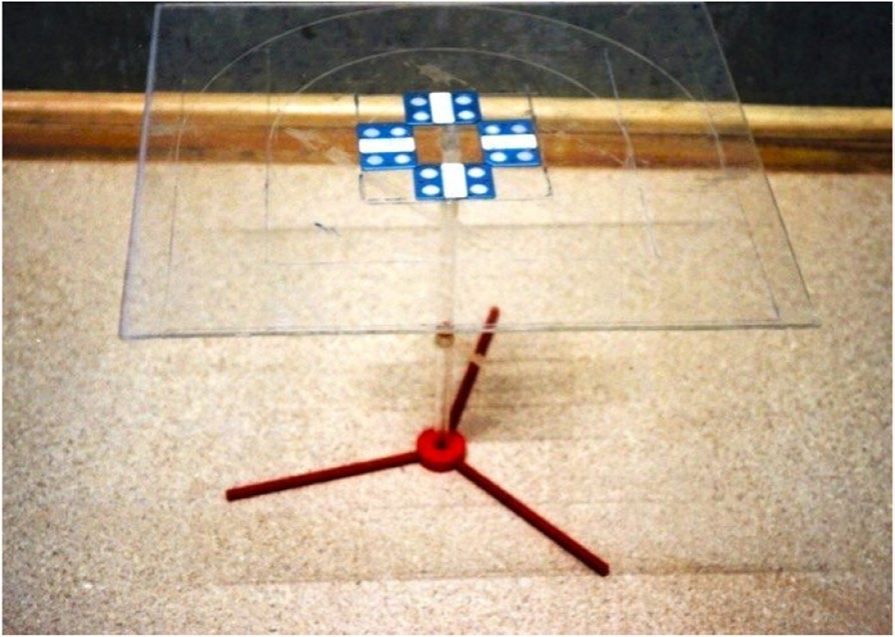
Calibration of TLD cards inside the 10 × 10 cm^2^ setup for dose measurement

TLD-100 (G1 cards), Harshaw, USA, in disc shapes as shown in Fig. 1 were used in this study. The TLD-100 (G1) cards contained two chips in duplicate. These cards were first annealed and calibrated against a known dose from Co-60 source at Secondary Standard Dosimetry Laboratories (SSDL), Pakistan Institute of Nuclear Science and Technology (PINSTECH), Islamabad Pakistan. The calibration factor for each card is determined. The detectors were again annealed and irradiated from Co-60 teletherapy unit (Theratron Phoenix) at the Institute of Radiotherapy and Nuclear Medicines (IRNUM), Peshawar, Pakistan. The irradiation conditions were (a) field size of 10 × 10 cm^2^ and (b) SSD of 80 cm. The radiation and reading time were fixed to be 48 h after irradiation in order to minimize the fading effect of the cards.

**Fig. 2:**
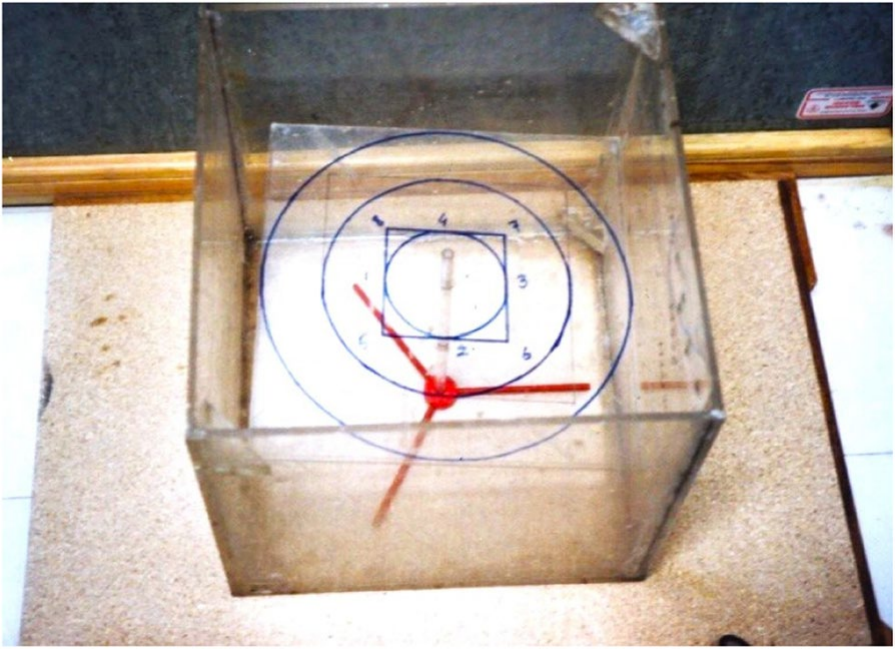
Isocentric circles where TLD cards are placed outside the radiation field in the phantom

Dimensions of the cards were 3.1 mm × 3.1 mm × 0.89 mm and were encapsulated between two sheets of Teflon of 0.06 mm thickness. The cards of 8800 series manufactured with the card identification number in bar code format. A dedicated water phantom of dimension 35.5 × 36 × 36.5 cm^3^ with acrylic disc and isocentric tracks (provided by IAEA) [45] for TLD-100 (G1 cards) placement and radiation exposure was used for taking the data, as shown in the Fig. 2.

The reading of the TLD-100 (G1 cards) was carried out at Radiation Dosimetry Group (RDG) PINSTECH using a fully automatic and computerized thermoluminescence dosimetry workstation 8800 [46]. Thirty TLD-100 (G1) cards were annealed in Health Physics Division (HPD), PINSTECH. A total of 30 TLD-100 (G1 cards) were used; among these, only 20 random TLD-100 (G1 cards) were chosen in the experiment.

TLD-100 cards were placed in circular tracks of radii 10, 15, and 18 cm in such a way that in the first and second track there were 8 cards at 45° intervals. However, the outer third track contain 4 cards at 90° interval with all circular tracks outside radiation field(8 + 8 + 4) as shown in Fig. 2.

This assembly was placed on the surface of water phantom and the annealed cards were exposed to a dose of 2000 mGy from Co-60 gamma rays [46] in succession at a depth of 5 cm in water phantom. The calibration factor was calculated by Eq. 1.

1 $$\mathrm{CF}=\frac{\mathrm{Dose}\;\mathrm{in}\;\mathrm{mGy}}{\mathrm{Response}\;\mathrm{in}\;\mathrm{nC}}$$

The exposed TLD-100 (G1 cards) was read out by taking four readings at each depth. In the whole experimental work, first TLD-100 (G1 cards) were read out and then again annealed, i.e., dual annealing was done, in order to avoid any residual peaks. In present work, the time interval of 48 h was fixed between the exposures and read out of TLD-100 (G1 cards, to maintain consistency in readings), using TRS-398 protocols [47]. To select the most reliable chip for the project work, F-test (statistical tool to compare the variance of two samples or the ratio of variances between multiple samples) was applied to check the most accurate chip response and found that the results of the chip-1 were more consistent than chip-1 [48]. Also, chip to chip calibration factor was available; therefore, it was better to use individual calibration factor for each chip-1. The PDs were measured for Co-60 beam at specified depths of 5, 10, and 15 cm outside the radiation beam at distances of 5, 10, and 13 cm away from the radiation field edge using TLD-100 (G1 cards). The isotropic distributions were confirmed and scattered to primary dose ratios were also estimated at the mentioned depths and distances from the radiation field edge in water phantom.

## Results

**Fig. 3:**
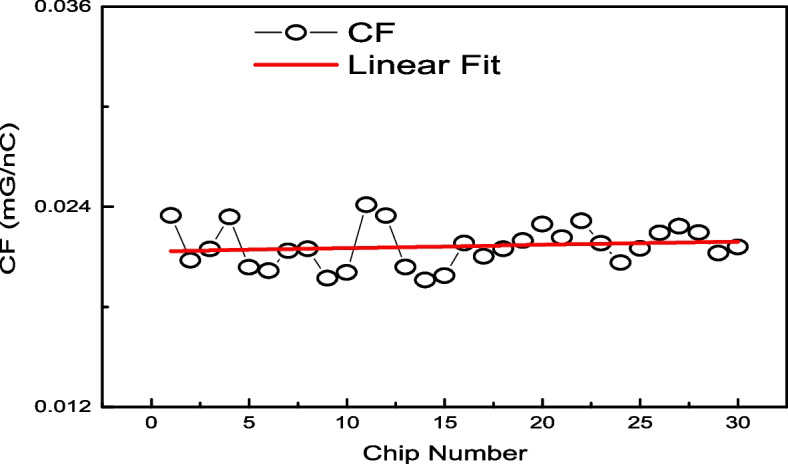
Chip-1 calibration factors and its deviation

Calibration factor for all chip-1 along with mean, standard deviation (SD), co-efficient of variation (COV), and mean deviation (MD) are shown in Table 1 and Fig. 3.

**Table 1:** Mean, standard deviations, coefficient of variation, and calibration factors for all chips (chip-1)

S.No	CF	TLD ID	S.No	CF	TLD ID
1	0.023479	423	**16**	0.021831	474
2	0.020821	424	**17**	0.02103	478
3	0.021465	425	**18**	0.021476	479
4	0.023394	426	**19**	0.021981	481
5	0.020378	427	**20**	0.022964	482
6	0.020165	428	**21**	0.022157	483
7	0.021368	429	**22**	0.023163	484
8	0.021482	430	**23**	0.02182	485
9	0.019722	431	**24**	0.020663	487
10	0.020069	437	**25**	0.021514	488
11	0.024131	439	**26**	0.022444	489
12	0.023471	468	**27**	0.022842	491
13	0.020391	469	**28**	0.022457	492
14	0.019611	470	**29**	0.021234	493
15	0.019883	471	**30**	0.021595	495
Mean			0.021633		
SD			0.001236		
MD			0.00173		
CV			5.7%

**Fig. 4:**
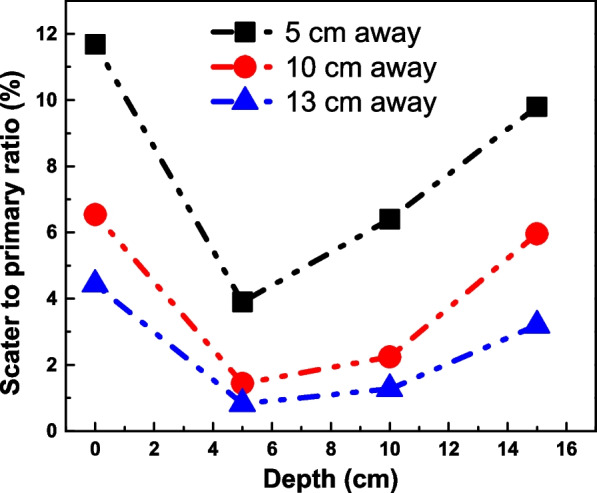
The scatter to primary doses ratios at various depths and various distances from the radiation field edge

The mean CF, SD, MD, and COV for all chip-1 are 0.021633, 0.001236, 0.00173, and 5.7% respectively. The scatter to primary doses ratios at surface 0, 5, 10, and 15 cm depths and away from the radiation field at distances of 5, 10, and 13 cm as shown in Table 2 and Fig. 4.

**Table 2:** Scatter to primary doses ratios at various depths along the various distances from the radiation field edge

Depth (cm)	Scatter/primary (ratio) 5 cm away	Scatter/primary (ratio) 10 cm away	Scatter/primary (ratio) 13 cm away
0	11.69%	6.54%	4.43%
5	3.9%	1.44%	0.83%
10	6.4%	2.237%	1.27%
15	9.8%	5.96%	3.2%

**Fig. 5:**
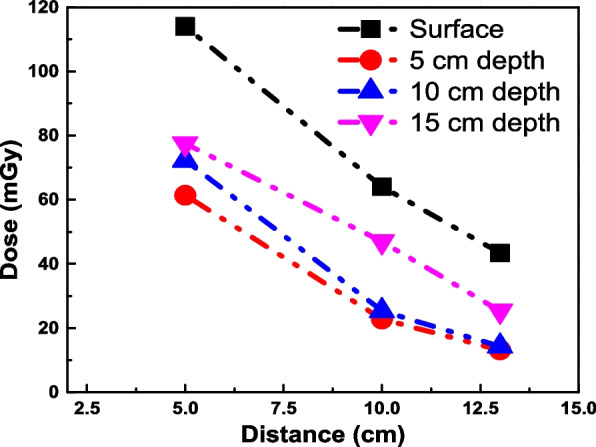
PDs at various depths and various distances away from the radiation field edges at constant SSD

PDs at various distances of 5, 10, and 13 cm away from radiation field edge along various depths of 0, 5, 10, and 15 cm with fixed SSD are shown in Table 3 and Fig. 5.

**Table 3:** PDs at varying depths and distances away from the radiation field edge at constant SSD

Distance	**Surface(0 depth) dose (mGy)**	**5 cm depth dose (mGy)**	**10 cm depth dose (mGy)**	**15 cm depth dose (mGy)**
5	114.028	61.36	72.71	77.5
10	64.09	22.76	25.36	46.93
13	43.406	13.095	14.25	25.3

## Discussion

Radiation therapy involves the delivery of a prescribed dose to the target volume, sparing the surrounding healthy organs and tissues as much as possible. Traditional techniques that ensure the prescribed dose gets delivered to the target volume include treatment planning systems and water/humanoid phantom studies. These dose monitoring methods are done before the radiation treatment for radiation dose delivery verification as a quality assurance tool and may reflect the radiation dose delivered during treatment. In the current study, dose was measured in the water phantom using TLD-100 (G1 cards) as detectors out of the irradiated field as PDs. The measured PDs provides dose distribution in the homogenous medium outside the irradiated volume; therefore, it estimates dose to the OAR in radiotherapy patients. The PDs along the horizontal distance showed strong dependency on the distance from the radiation field edge and follows an exponential decrease along the surface and can be assumed to dose received on the surface. The dose at depth in water phantom shows the dose distribution inside the scattering medium. The PDs at 5, 10, and 13 cm away in circular track confirmed the isotropic distribution of radiation dose from Co-60 source along the depth and surface. The scattered to primary dose ratios were 11.69%, 6.53%, and 4.43% at 5, 10, and 13 cm away from the treatment field edge respectively on the surface. % PD is higher on the phantom surface. The surface dose was observed high due to contribution from all the scattering components of the Co-60 machine [49, 50]. The PDs recorded at 5 cm depth and 5, 10, and 13 cm away from the field edge was the least. Scattered to primary dose ratios at these positions were 3.89%, 1.44%, and 0.83% respectively. Internal scattered radiation is the predominant source of PDs. The depth dependence is determined by the attenuation of the primary photons; the reason of these least PDs is least scattering due to contribution from the collimator, phantom material, and other sources such as leakage and penumbra of Co-60 source having insufficient energy to reach the detectors at these points. The second reason behind this behavior is also the dominant forward scattering of cobalt-60 gamma rays [49, 50] and least scattering towards the sides at this depth. Also, the behavior of the PDs around the source was noted as isotropic. The PDs recorded at 10 and 15 cm depths showed clear increase in PDs than at 5 cm depth and is approximately doubled and tripled, respectively. The PDs decreases almost exponentially with the increase of distance from the field edge at these depths. The scatter to primary dose ratios at 5, 10, and 13 cm away from the radiation field edge at these depths (10 and 15 cm) were 6.4% and 9.8%, 2.25% and 5.95%, and 1.26% and 3.21%, respectively as shown in Table 2. The change in trend/increase in the PDs at these depths have several reasons like (a) scattering medium; (b) increased irradiated volume; (c) increase in penumbra; (d) increase SAD as SSD was fixed in the study, i.e., inverse square factor; (e) less forward scattering of Co-60 beam; and (f) increase inside and back scattering. So, all these factors contributed in increase in scattered dose [49].

This experimental study confirmed that the dose distribution is isotropic, so the PDs at any depths and distance can be estimated by applying proper interpolation that will be helpful to estimate the absorbed dose to OARs in RT of cancer patients. Accordingly, that will be used in retrospective/prospective studies examining possible correlation between RT recommended dose and secondary cancer incidence/risk. It also leads to an accurate assessment of out-of-field dose/PD necessary to estimate the risk of second cancer after radiotherapy and the damage to the organs at risk surrounding the planning target volume.

## Conclusion

It is concluded that measured PDs in water phantom as a homogenous medium estimates the absorbed dose to OARs in radiotherapy of cancer patients. The PDs as a function of distance and depths showed strong dependency on radiation dose given, depth in tissue, distance from the target volume, SAD, inverse square factor, penumbra, irradiated volume, collimator scattering, leakage from the source housing, and scattered radiation in the tissue. The increase of PDs along the surface and depth beyond 5 cm do not recommend the use of Co-60 unit for treatment of superficial and deep-seated tumors at greater depths, i.e., beyond 5 cm using single field. It is suggested that the PDs can be minimized by using the isocentric technique and multiple fields with beam weighting as these techniques controls sources of scattered radiation like inverse square factor, given the dose and effect of penumbra up to some extent especially in patients having long-term surveillance expectancy. Special attention is required for tumors near moving organs like the lungs and diaphragm.

## Data Availability

The datasets used and analyzed in this study are available from the corresponding author upon reasonable request.

## Abbreviations

SD: Standard deviation
COV: Coefficient of variation
MD: Mean deviation
SSD: Source to surface distance
SAD: Source to axis distance
PD: Peripheral dose
CF: Calibration factor
TLD: Thermoluminescent detector
